# On the optimal number of dose‐limiting shells in the SBRT auto‐planning design for peripheral lung cancer

**DOI:** 10.1002/acm2.12983

**Published:** 2020-07-23

**Authors:** Yanhua Duan, Wutian Gan, Hao Wang, Hua Chen, Hengle Gu, Yan Shao, Aihui Feng, Yanchen Ying, Xiaolong Fu, Chenchen Zhang, Zhiyong Xu, Ning Jeff Yue

**Affiliations:** ^1^ Department of Radiation Oncology Shanghai Chest Hospital Shanghai Jiao Tong University Shanghai China; ^2^ Shcool of Physics and Technology University of Wuhan Wuhan China; ^3^ Department of Radiation Oncology Rutgers Cancer Institute of New Jersey Rutgers University New Brunswick NJ USA

**Keywords:** auto‐planning, dose‐limiting shell, peripheral lung cancer, SBRT

## Abstract

**Purpose:**

The number of dose‐limiting shells in the optimization process is one of the key factors determining the quality of stereotactic body radiotherapy (SBRT) auto‐planning in the Pinnacle treatment planning system (TPS). This study attempted to derive the optimal number of shells by evaluating the auto‐plans designed with different number of shells for peripheral lung cancer patients treated with SBRT.

**Methods:**

Identical treatment technique, optimization process, constraints, and dose calculation algorithm in the Pinnacle TPS were retrospectively applied to 50 peripheral lung cancer patients who underwent SBRT in our center. For each of the patients, auto‐plans were optimized based on two shells, three shells, four shells, five shells, six shells, seven shells, eight shells, respectively. The optimal number of shells for the SBRT auto‐planning was derived through the evaluations and comparisons of various dosimetric parameters of planning target volume (PTV) and organs at risk (OARs), monitor units (MU), and optimization time of the plans.

**Results:**

The conformity index (CI) and the gradient index (GI) of PTV, the maximum dose outside the 2 cm of PTV (D_2cm_), D_max_ of spinal cord (SC_max_), the percentage of volume of total lung excluding ITV receiving 20 Gy (V20) and 10 Gy (V10), and the mean lung dose (MLD) were improved when the number of shell increased, but the improvement became not significant as the number of shell reached six. The monitor units (MUs) varied little among different plans where no statistical differences were found. However, as the number of shell increased, the auto‐plan optimization time increased significantly.

**Conclusions:**

It appears that for peripheral lung SBRT plan using six shells can yield satisfactory plan quality with acceptable beam MUs and optimization time in the Pinnacle TPS.

## 1 INTRODUCTION

With the advancement of radiotherapy technologies, precision radiotherapy becomes a reality, and its demand for the target dose coverage conformity and organs at risk (OARs) sparing has also become more stringent, making the design of treatment plans complex and time‐consuming. The inverse treatment planning has become one of the major treatment planning techniques nowadays, and it can help to achieve dose optimization through well‐defined optimization constraints with satisfactory plan quality.^1^, ^2^, ^3^ In the inverse planning process, dose‐limiting shells are often introduced and used to control the dose gradient outside the target to an acceptable level. The main mechanism of the dose‐limiting shells is to generate several artificial three‐dimensional rings or shell structures at certain distances outside the target, and the doses of these artificial structures are used as part of the optimization constraints to limit or reduce the doses of normal tissues surrounding the target to achieve desired dose gradient outside the target without compromising the dose coverage to the target while sparing as much normal tissues as possible. Thus, the roles of these shells can play important roles in determining the quality of a plan when the inverse planning technique is used.^4^

Several groups conducted studies on the planning strategy using the dose‐limiting shells. Cao et al.^4^ studied the effects of dose‐limiting auto‐shells on pancreatic cancer radiotherapy treatment plans based on the robotic radiosurgery system. Sharfo et al.^5^ used three dose‐limiting shells in the optimization of cervical cancer planning to limit the conformability of the target. Voet et al.^6^ used four shells with different restricted doses around the target of prostate cancer patients to improve the quality of the treatment plans.

Most of those studies on the dose‐limiting shells focused on the conventional fractionated radiotherapy. For stereotactic body radiotherapy (SBRT), it is almost always required to have high dose gradients around the target volumes while limiting the doses to OARs as low as possible. To date, few studies have been conducted to investigate the effects of number of the dose‐limiting shells on the qualities of the SBRT treatment plans. In addition, as the auto‐planning system is being more introduced to the routine clinical use and its process is different from the conventional inverse planning in terms of the optimization condition setting, there is a need to conduct investigations into the use of the shells in the automatic planning for SBRT treatment to determine their impacts on the plan qualities, beam output amount (MUs), and planning efficiency so that an optimal number of shells in the SBRT auto‐planning can be derived.

Our institution is a dedicated thoracic tumor center, and has a wealth amount of peripheral lung cancer cases and treatment experiences. This study implemented different numbers of shells in the auto‐plans for peripheral lung cancer patients treated with SBRT to evaluate the impacts of number of the dose‐limiting shells on the dose coverage of the targets and the dose sparing of OARs, to explore the optimal number of shells in the Pinnacle TPS for peripheral lung cancer patients treated with SBRT. The investigational intent is to provide guidance for the optimal settings and number of the shells during the auto‐planning process of SBRT peripheral lung cancer treatments.

## 2 MATERIALS AND METHODS

### 2.A. Patients collection

The 50 inclusion cases were the patients who had peripheral NSCLC that could not or would not be resected, and two or more radiotherapists agreed that they were suitable for SBRT. This study selected 50 (29 men and 21 women) patients treated in our center from January 2016 to September 2018. The age of the patients ranged from 38 to 76 (median age 66 yr and mean age 65 yr). Regarding the location of the targets, there were 10 cases of upper left lobe, 12 cases of lower left lobe, 11 cases of upper right lobe, 7 cases of right middle lobe, and 10 cases of right lower lobe. The volume of ITV ranged from 5.05 to 6.92 cm^3^ (mean volume 5.81 cm^3^ and std 0.56 cm^3^), the volume of PTV ranged from 19.64 to 27.23 cm^3^ (mean volume 22.50 cm^3^ and std 1.94 cm^3^), and clinical stage was T_1_N_0_M_0_. The prescription dose was 50 Gy by five fractions or 50 Gy by four fractions depending on tumor size, location, and patient's physical condition. When the study began, all selected patients signed informed consents and completed radiotherapy. And this study was approved by the native Ethics Committee (the committee's reference Number: KS1863).

### 2.B. Structure contouring and auto‐plan designing

Treatment targets and OARs were delineated by experienced radiation oncologist. Planning target volumes (PTV) were obtained by expanding 0.5 cm of internal target volume (ITV) in three dimensions. All structures were reviewed and approved by at least one other experienced radiation oncologists before being used for planning design.

**Fig. 1:**
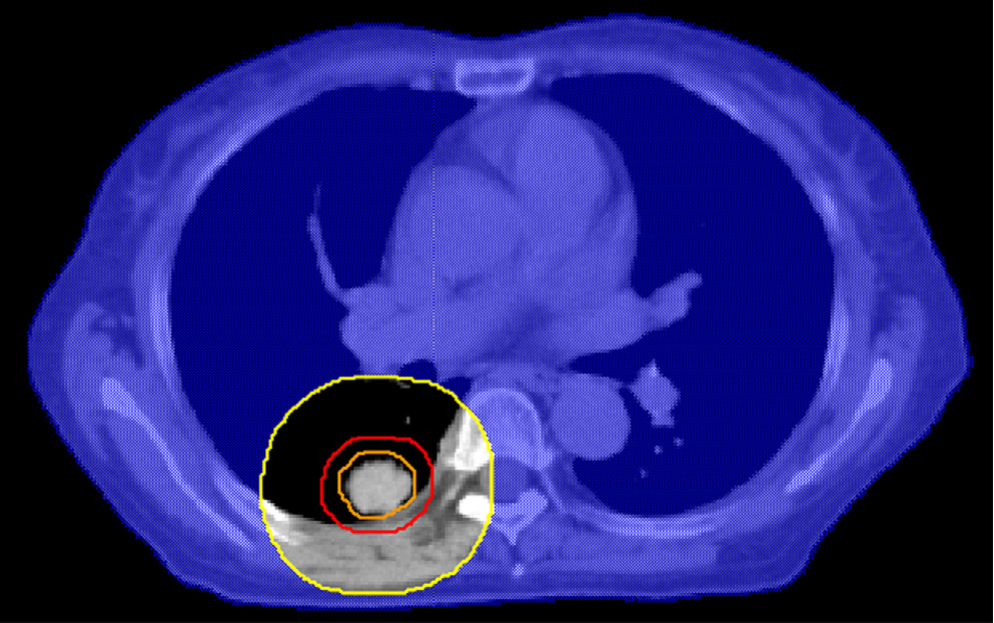
Cross‐sectional view of the 2‐cm shell (orange: internal target volume, red: planning target volumes (PTV), yellow: auxiliary structure after 2‐cm expansion of PTV, blue‐shaded area: 2‐cm shell obtained by subtracting auxiliary structure from body).

The generation of the dose‐limiting shells is briefly described as follows. First, the PTV was expanded to a certain boundary to generate an intermediate structure, and the intermediate structure was then subtracted from the body to generate a shell (e.g., see Fig. 1).

A prewritten script was used to generate eight shells for each patient, and seven treatment plans were designed based on different numbers of shells (Table 1), then each shell was restricted by a maximum dose (Table 2). All settings were the same between different plans of a same patient except for the number of shells. In the planning process, the dosimetric coverage requirements of the target and the dose constraints of the OARs followed the recommendations of radiation oncology working group (RTOG) 0915^7^ or stricter standards. Specifically, 100% prescription dose is prescribed to cover no <95% of the PTV. The percentage of volume of total lung excluding ITV receiving 10 Gy (V10) and 20 Gy (V20) must be <25% and 10%, respectively. The maximum dose of spinal cord (SC_max_) must be <20 Gy.

**Table 1:** The details of different shell groups.

	Distance from PTV margin to shell (mm)							
	1st shell	2nd shell	3rd shell	4th shell	5th shell	6th shell	7th shell	8th shell
Two‐shell plan	8	15	–	–	–	–	–	–
Three‐shell plan	5	8	15	–	–	–	–	–
Four‐shell plan	3	5	8	15	–	–	–	–
Five‐shell plan	3	5	8	15	25	–	–	–
Six‐shell plan	3	5	8	11	15	25	–	–
Seven‐shell plan	3	5	8	11	15	20	25	–
Eight‐shell plan	3	5	8	11	15	20	25	30

Abbreviations: PTV, planning target volume.

**Table 2:** Maximum dose constraints of each shell.

	Maximum dose constraints (Gy)							
	1st shell	2nd shell	3rd shell	4th shell	5th shell	6th shell	7th shell	8th shell
Two‐shell plan	20	14	–	–	–	–	–	–
Three‐shell plan	25	20	14	–	–	–	–	–
Four‐shell plan	28	25	20	14	–	–	–	–
Five‐shell plan	28	25	20	14	9	–	–	–
Six‐shell plan	28	25	20	17	14	9	–	–
Seven‐shell plan	28	25	20	17	14	11	9	–
Eight‐shell plan	28	25	20	17	14	11	9	7

Following the principle that the fields should be closest to the target, all the treatment plans were IMRT and were designed using the auto‐planning (AP) module of the Pinnacle TPS (V9.10,Philips Radiation Oncology Systems, Fitchburg, WI, USA) with 10 or more 6 MV coplanar beams for an Edge^TM^ linear accelerator (Varian Medical Systems, Palo Alto, CA), and the angular interval of the fields was 15°. For the target optimization goals, PTV was set to the prescribed dose. For the OAR optimization goals, each shell was given the maximum dose constraint with a high priority according to the settings in Tables 1 and 2. Other normal tissues were not restricted, because when the shell dose reaches the established requirements, the dose of OARs can meet the clinical requirements even if it is close to the target. The direct machine parameter optimization (DMPO) algorithm and the collapsed cone convolution (CCC) algorithm were used for plan optimization and dose calculation, respectively.

It should be noted that the AP in the Pinnacle TPS refers to the automation of the plan optimization. Before the automatic optimization, the plan designer needs to add the fields and the optimized goals to the plan manually. The automatically optimized goals include the target dose for PTV, the constraints for OARs or auxiliary structures. For the lung SBRT plans in this paper, the process of adding the automatically optimized goals includes setting a prescription dose of 50 Gy for PTV and setting the maximum dose constraints listed in Table 2 for each shell. This step is also completed by prewritten scripts. Subsequently, the AP module is used to perform automatic optimization of the plan.

### 2.C. Evaluation index of Plan quality

The evaluation criteria of the target included CI, GI, D_2cm_. For OARs, the evaluated parameters included SC_max_, V20, V10, and the mean lung dose (MLD). In addition, the impacts of number of the shells on the plan total MUs and optimization times were investigated in the study.

The target CI was computed as:^8^, ^9^

$$\text{CI}=V_{T,\text{Rx}}^{2}/\left(V_{T}\times V_{\text{Rx}}\right)$$

where V_T,Rx_ is the volume of target receiving a dose equal to or greater than the prescription dose, V_T_ is the target volume, and V_Rx_ is the volume receiving a dose equal to or greater than the prescription dose. The range of CI is from 0 to 1, where CI = 1 indicates that the conformability is the best, and CI = 0 indicates that there is no conformality of target dose coverage.

The GI is calculated as:^10^, ^11^

$$\text{GI}=V_{50\%\text{Rx}}/V_{\text{Rx}}$$

where V_Rx_ is the volume receiving a dose equal to or greater than the prescription dose, V_50%Rx_ is the volume receiving a dose equal to or greater than half the prescription dose. A lower value of GI represents a faster dose fall‐off in normal tissue from the target.

### 2.D. Statistical analysis

Statistical analysis was performed using SPSS 22.0（SPSS Inc., Armonk, NY）. The one‐way repeated measures analysis of variance (ANOVA) was used for comparisons between seven plans with different shells. Bonferroni test was performed for comparisons between any two plans when result was significant after one‐way repeated measures ANOVA. A *P *< 0.05 was considered statistically significant.

## 3 RESULTS

### 3.A. Evaluations of PTV in different plans

**Fig. 2:**
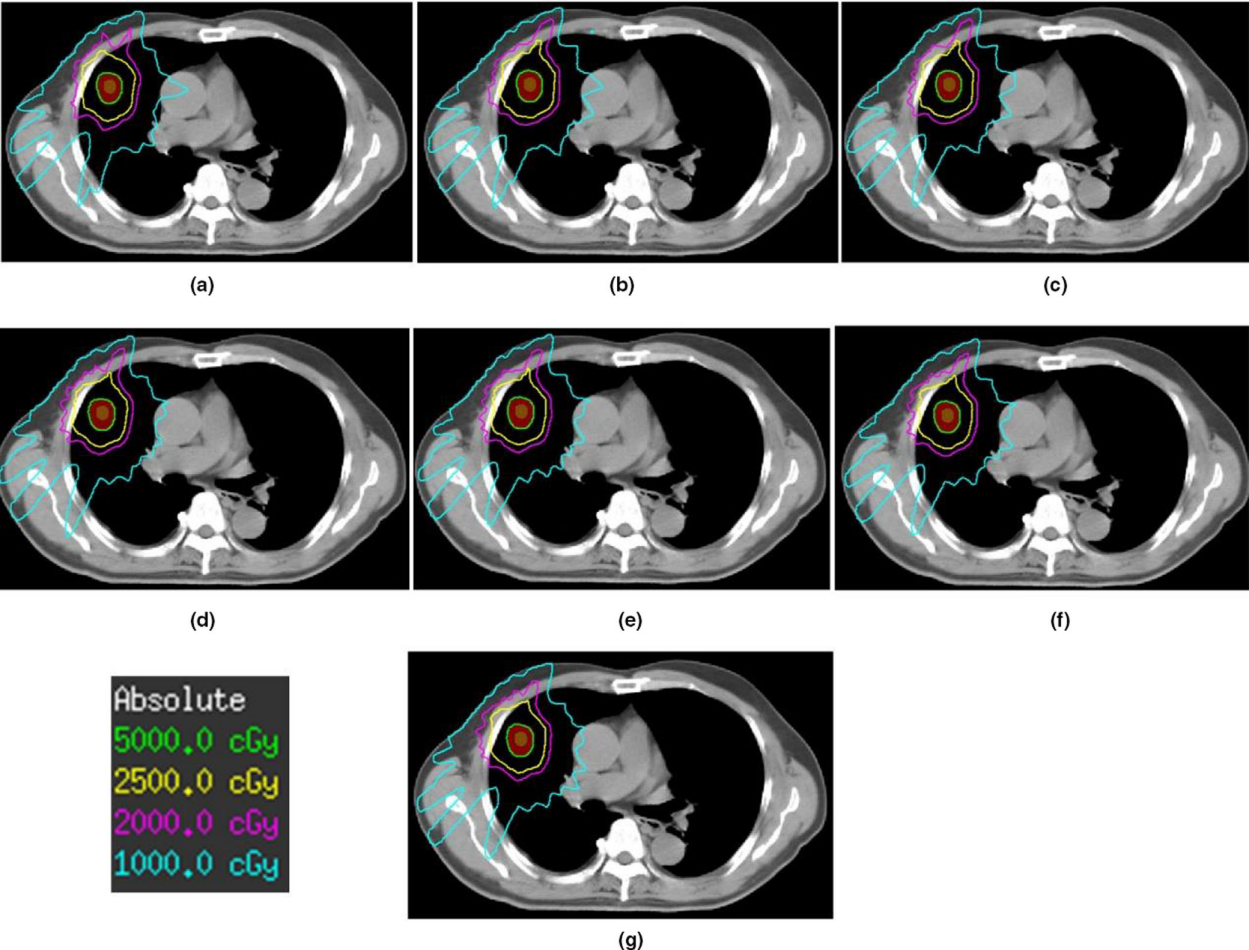
Dose distribution for different shell plans (a: two‐shell, b: three‐shell, c: four‐shell, d: five‐shell, e: six‐shell, f: seven‐shell, and g: eight‐shell).

Figure 2 shows an example of the dose distribution between plans with different number of shells of a same patient. The different plans of the same patient exhibited little visual differences in dose distribution. Table 3.a lists the dosimetric parameter comparisons of all the plans for PTV. For a same patient, CI, GI, and D_2cm_ of PTV showed a trend of gradual improvement. CI was closer to 1 (0.887–0.891), GI was lower (5.194–4.725), and the D_2cm_ was lower (29.200–26.066 Gy) with the increase in number of the shells after the optimization.

**Table 3:** Evaluations of different plans (mean and range).

Criteria	a. Parameters for PTV			b. Parameter for OARs				c. MU and optimization time	
	CI	GI	D_2cm_ (Gy)	SC_max_ (Gy)	V20 (%)	V10 (%)	MLD (Gy)	MU	Time(s)
Two‐shell	0.887 (0.833–0.913)	5.194 (4.362–6.177)	29.200 (21.956–38.463)	10.413 (2.826–18.789)	3.474 (1.523–6.930)	8.019 (3.730–20.949)	2.916 (1.455–4.654)	2154.000 (1586–3394)	296.800 (232–365)
Three‐shell	0.887 (0.839–0.915)	4.995 (4.144–5.816)	27.311 (21.126–34.384)	10.224 (2.819–18.232)	3.357 (1.494–6.754)	7.773 (3.609–17.587)	2.868 (1.485–4.622)	2160.820 (1574–3423)	337.400 (266–421)
Four‐shell	0.888 (0.834–0.917)	4.889 (4.155–5.446)	26.811 (20.891–31.335)	10.086 (2.910–17.255)	3.280 (1.468–6.738)	7.683 (3.589–18.688)	2.830 (1.452–4.585)	2168.980 (1598–3225)	390.000 (319–470)
Five‐shell	0.890 (0.840–0.914)	4.837 (4.170–5.260)	26.564 (21.422–28.358)	9.890 (3.028–16.897)	3.237 (1.452–6.661)	7.627 (3.606–18.483)	2.821 (1.469–4.556)	2174.740 (1622–3395)	433.000 (363–532)
Six‐shell	0.890 (0.859–0.952)	4.789 (4.006–5.044)	26.281 (20.754–28.639)	9.769 (2.829–15.216)	3.223 (1.432–6.603)	7.607 (3.557–18.408)	2.817 (1.457–4.571)	2185.560 (1622–3223)	480.400 (401–593)
Seven‐shell	0.891 0.839–0.909)	4.741 (3.987–5.031)	26.169 (20.074–27.522)	9.666 (3.393–16.386)	3.220 (1.462–6.568)	7.576 (3.583–18.380)	2.812 (1.422–4.543)	2190.400 (1608–3235)	524.600 (435–652)
Eight‐shell	0.891 (0.852–0.944)	4.725 (4.004–5.027)	26.066 (19.040–28.001)	9.552 (3.397–15.811)	3.214 (1.482–6.539)	7.564 (3.706–17.321)	2.810 (1.452–4.547)	2192.840 (1597–3265)	566.200 (478–691)
p‐value*	0.529	<0.001	0.024	<0.001	<0.001	<0.001	<0.001	0.531	<0.001

Abbreviations: CI, conformity index; D_2cm_, the maximum dose outside the 2 cm of target; GI, gradient index; MLD, the mean lung dose; MU, monitor units; OARs, organs at risk; PTV, planning target volume; SC_max_, the maximum dose of spinal cord; V10, V10 of total lung; V20, V20 of total lung.

* *P*‐value of one‐way repeated measures ANOVA.

### 3.B. Evaluations of OARs in different plans

Table 3.b lists the dosimetric parameter comparisons for OARs. For a same patient, SC_max_ (10.413–9.552 Gy), V20 (3.474–3.214%), V10 (8.019–7.564%), and MLD (2.916–2.810 Gy) all showed a trend of gradual improvement (decrease) with the increase in number of the shells after the optimization.

### 3.C. MU and optimization time in different Plans

Table 3.c tabulates the mean MUs per fraction of the selected 50 cases and optimization times in different plans. The mean MUs (2154–2193) and optimization times (297–566 s) were both increased with the increase in number of the shells.

### 3.D. The result of statistical analysis

The results of one‐way repeated measures ANOVA test showed that there were significant differences in GI, D_2cm_, SC_max_,V20, V10, MLD, and optimization time among the seven groups (*P* < 0.05), but the statistical differences were not found in CI and MUs among the seven groups (*P* > 0.05).

Subsequently, Bonferroni test was performed to compare any two plans for the parameters which result was significant after one‐way repeated measures ANOVA. For GI, V20, and V10, any group of the plans optimized with five or less shells was significantly different from at least one group of the plans optimized with six or more shells (*P* < 0.05), and there was no difference between any two plans optimized with six shells or more (*P* > 0.05). Similarly, for SC_max_ and MLD, no statistical differences were found between any two groups of plans optimized with five or more shells (*P* > 0.05). For D_2cm_, there were no statistical differences between any two groups of plans with three or more optimization shells (*P* > 0.05). Overall, there were no statistical differences between any two groups of plans with six or more optimization shells for all dosimetric parameters.

Unlike the dosimetric evaluation criteria, there were significant differences between any two plans in the optimization time (*P* < 0.05).

### 3.E. Determination of the optimal number of the dose‐limiting shells

Statistical results showed that there was no difference in CI and MU. It indicated that the CI and MUs were similar for plans with different number of shells, thus those two parameters could not provide decision‐making information for the selection of the optimal shell number.

**Fig. 3:**
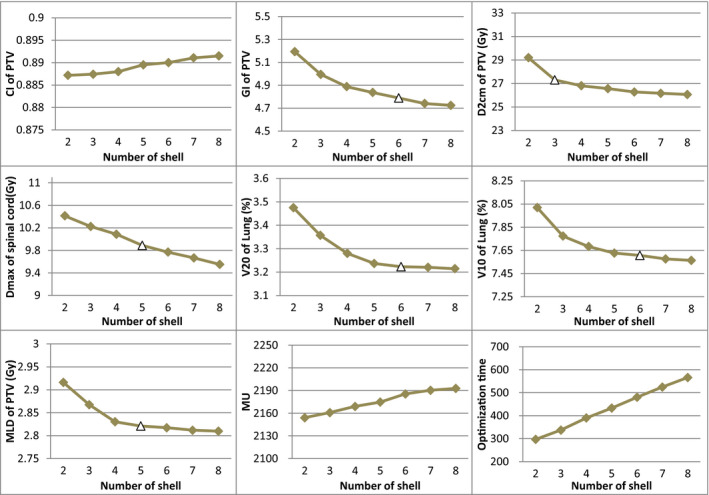
Line charts of criteria in different plans (The triangle (△) represents optimal dose‐limiting shells).

Figure 3 presents the trends of the evaluated parameters as the number of the shells increased. It is evident that all the dosimetric parameters improved as the number of the shells increased. For GI, V20, and V10, any plan with five or less optimization shells is different significantly from at least one plan with six optimization shells or more (*P* < 0.05), but there was no difference between any two plans optimized with six shells or more (*P* > 0.05). It could be inferred that when the number of the shells was five or less, the auto‐plan optimization system was able to improve plan quality by non‐trivial margin with the increase in the shell number. However, as the number of the shells further increased to six, the plan quality improvement became limited as the number of shells increased even though the optimization became significantly longer. Therefore, for the three parameters of GI, V20, and V10, the optimal number of shells should be six for peripheral lung SBRT auto‐plan. Similarly, the optimal number of shells should be five for SC_max_ and MLD, and three for D_2cm_. In Fig. 3, we marked the optimal shell number with the hollow triangle symbol for each evaluation parameter, except for CI and MU, in which no significant differences were found in seven groups of plans, and optimization time, in which there were significant differences between any two groups of plans.

Based on the trends and statistical results of each evaluated parameters, it could be concluded that when the shell number was increased to six, the improvement in all dosimetric parameters was no longer significant with the increase in shells, and the optimal number of shells seems to be six for peripheral lung SBRT using the Pinnacle TPS.

## 4 DISCUSSIONS

The inverse planning is one of the main methods for high‐precision radiotherapy planning. When using the auto‐planning system, the control of the target dose gradient is highly dependent on the constraints of the dose‐limiting shells. The number of shells used in the process is one of the key factors determining the quality of the plan.^4^ Previous studies on the roles of the dose‐limiting shells were mostly directed to conventional dose radiotherapy based on manual planning. However, SBRT requires a higher dose gradient around the target volume and restricted dose constraints to OARs than conventional therapy. In addition, as the auto‐planning becomes more popular and is able to achieve better plan quality than the manual planning process,^12^, ^13^, ^14^ and its optimization condition setting are different from manual planning, it becomes important to understand the roles of number of the dose‐limiting shells in the SBRT auto‐planning process.

In this paper, a range of numbers of shells were implemented in the auto‐planning for peripheral lung cancer patients treated with SBRT. By evaluating the quality of different plans, the optimal number of shells was obtained for the first time in the SBRT auto‐planning of peripheral lung cancer using the Pinnacle TPS system.

In the results of this paper, the standard deviation comes for the greater part from systematic deviations between patients. The variance of dosimetry in different plans optimized by the same number shells only reflects the difference such as tumor size, location, and patient's physical condition. This variance is meaningless for our result analysis, so we did not list the standard deviation in the tables and figures.

As shown in the result of statistical analysis, there were no statistical differences in CI and MU among the seven groups of plans with different shells (*P* > 0.05). As far as those two parameters were concerned, there were no differences in plans optimized with different number of shells, thus these two indexes could not be used as the criteria to judge the quality of different plans.

As can be seen from Table 3 and Fig. 3, the quality of the plan gradually improved as the number of shells increased. For the dosimetric parameters with statistical differences (GI, V20, V10, SC_max_, MLD, and D_2cm_), if a group of plans differed significantly from another one or more groups with more shells, it indicated that this group of plans were not the optimal, and the quality of plans would significantly improve if more shells were used. When there were no differences between any two in several groups of plans, it indicated that the qualities for these groups of plans are almost the same, and the minimum quantity of shells in these plans could be considered as the optimal number for shells. The optimal shell number was different for different evaluation parameters (six for GI, V20 and V10, five for SC_max_ and MLD, three for D_2cm_), but there was no difference between any two groups of plans optimized with six or more shells for all dosimetric parameters (*P* > 0.05). Thus the plan quality had no further improvement as the shell number increasing when the number of shells reached six. In other words, adding additional shells beyond six had little effect on the quality of the plan using the Pinnacle TPS.

Since the generation of the shells and the setting of optimization conditions for different plans were all done through prewritten scripts during the planning design, the preparation time before the automatic optimization of different shell plans was similar. So we did not count the time of the entire plan design, but only the automatic optimization time of different plans. As shown in Fig. 3, the plan optimization time increased almost linearly with the number of the shells, and the statistical results had proved the significance of the differences (*P* < 0.001). In addition, although there was no statistical difference in MUs, MUs increased with the increasing number of shells. More MUs indicated that both the planning complexity and the inconsistency between the planned dose and the delivered dose increased.^15^ Therefore, on the premise of obtaining satisfactory planning quality, it is very important to find a shell number as small as possible to save the planning design time and improve the work efficiency. When the number of shells is less than six, there are still some dosimetric indexes improved significantly with the increase of shells, but all dosimetric parameters for both the PTV and OARs exhibit relatively small improvement when the number of the shells was six or more, the optimal number of shells for peripheral lung SBRT planning using the auto‐plan module of Pinnacle TPS system appears to be justified to be six.

It is well known that CI is a common evaluation criterion in many clinics, but there was no difference in CI in our research results. According to our research, although there was no statistical difference in CI among the seven groups of plans with different shells, the values of CI were improved from the two‐shell plan to the six‐shell plan (0.887–0.890, see Table 2), indicating a tendency of getting better in CI as the shell number increasing. Other evaluation criteria (including GI, D_2cm_, SC_max_, V20, V10 and MLD) were significantly improved with the increase of shells when the number of shells was less than six. The mean plan optimization time of the six‐shell plans (480.4 s) was only 183.6 s (3.06 mins) more than that of two‐shell plans (296.8 s). Considering the improvement in the overall quality of the plan, the short increased optimization time is acceptable.

According to the conclusions we have drawn, we want to overview the specific operation process of the six‐shell planning using the Pinnacle TPS in this study. Firstly, six shells of different distances (see Table 1 for details) were generated for PTV. Then, fields were added to plan. And finally, optimization goals were set for the plan and automatic optimization was started. The details of beam arrangement and optimization criterion were descripted in ‘structure contouring and auto‐plan designing’ part.

It should be noted that all the cases selected for this study were peripheral lung cancer patients. Whether the conclusion drawn from this study is applicable to the auto‐planning process for targets located in other regions of lung needs to be further evaluated and confirmed. It should be also pointed that this study was conducted using the auto‐plan module of Pinnacle TPS system. The conclusion may be different if other modules or other TPS systems are used. The impact of the shell or ring on other planning systems must be re‐examined using methods similar to this study or other measures based on specific system characteristics.

To the best of our knowledge we investigated the optimal number of the dose‐limiting shells in SBRT automatic planning for peripheral lung cancer for the first time. It is an important basis for setting up dose‐limiting shells in SBRT automatic planning for peripheral lung cancer patients. However, the impacts of dose‐limiting constraints of the shells were not studied. In this particular study, the shell dose constraints were set based on years of experiences at our institution, and we did not find better dose constraints after repeated experiments. Moreover, the conclusion of dose gradient is similar to our setting in a recently published study on lung SBRT.^16^ In addition, there are few studies similar to our research, so the specific distance values of the shells cannot be referenced. The distances were 3, 5, 8, 11, 15, 20, 25, and 30 mm from PTV for the first shell to the eighth shell, which was also partly due to our years of experience. Our team also calculated the relationship between dose drop speed and the specific distance based on the conclusion obtained in Ref. [16] to give a reasonable distance of each shell.

## 5 CONCLUSION

Considering the planning quality and time benefit, the optimal number of the dose‐limiting shells appears to be six in the design of SBRT plan for the peripheral lung cancer using the Pinnacle TPS. When the number of shells is more than six, adding more shells does not significantly improve the quality of the plan, but significantly increase the optimization time.

## CONFLICT OF INTEREST

None.
